# Understanding Uganda’s early adoption of novel differentiated HIV treatment services: a qualitative exploration of drivers of policy uptake

**DOI:** 10.1186/s12913-023-09313-x

**Published:** 2023-04-05

**Authors:** Henry Zakumumpa, Japheth Kwiringira, Cordelia Katureebe, Neil Spicer

**Affiliations:** 1 School of Public Health, Makerere University, Kampala, Uganda; 2 Department of Sociology, Kyambogo University, Kampala, Uganda; 3 Ministry of Health, AIDS Control Program, Kampala, Uganda; 4 London School of Hygiene and Tropical Medicine, London, UK

**Keywords:** Antiretroviral therapy, HIV treatment, Differentiated service delivery, DSD, Case study, Health policy and systems, Uganda

## Abstract

**Background:**

Although differentiated service delivery (DSD) for HIV treatment was endorsed by the WHO in its landmark 2016 guidelines to lessen patients’ need to frequently visit clinics and hence to reduce unnecessary burdens on health systems, uptake has been uneven globally. This paper is prompted by the HIV Policy Lab’s annual report of 2022 which reveals substantial variations in programmatic uptake of differentiated HIV treatment services across the globe. We use Uganda as a case study of an ‘early adopter’ to explore the drivers of programmatic uptake of novel differentiated HIV treatment services.

**Methods:**

We conducted a qualitative case-study in Uganda. In-depth interviews were held with national-level HIV program managers (*n* = 18), district health team members (*n* = 24), HIV clinic managers (*n* = 36) and five focus groups with recipients of HIV care (60 participants) supplemented with documentary reviews. Our thematic analysis of the qualitative data was guided by the Consolidated Framework for Implementation Research (CFIR)’s five domains (*inner context, outer setting, individuals, process of implementation*).

**Results:**

Our analysis reveals that drivers of Uganda’s ‘early adoption’ of DSD include: having a decades-old HIV treatment intervention implementation history; receiving substantial external donor support in policy uptake; the imperatives of having a high HIV burden; accelerated uptake of select DSD models owing to Covid-19 ‘lockdown’ restrictions; and Uganda’s participation in clinical trials underpinning WHO guidance on DSD. The identified processes of implementation entailed policy adoption of DSD (such as the role of local Technical Working Groups in domesticating global guidelines, disseminating national DSD implementation guidelines) and implementation strategies (high-level health ministry buy-in, protracted patient engagement to enhance model uptake, devising metrics for measuring DSD uptake progress) for promoting programmatic adoption.

**Conclusion:**

Our analysis suggests early adoption derives from Uganda’s decades-old HIV intervention implementation experience, the imperative of having a high HIV burden which prompted innovations in HIV treatment delivery as well as outer context factors such as receiving substantial external assistance in policy uptake. Our case study of Uganda offers implementation research lessons on pragmatic strategies for promoting programmatic uptake of differentiated treatment HIV services in other countries with a high HIV burden.

## Background

Sub-Saharan Africa (SSA) has the highest HIV burden in the world. Of the 37 million people living with HIV in the world, 20.6 Million (56% of the total) come from this region alone [1].

Within SSA, Eastern and Southern Africa has the highest HIV prevalence [1]. Four countries in particular, South Africa, Eswatini, Botswana and Lesotho, contribute the highest number of people on antiretroviral therapy (ART) worldwide [1].

The majority of countries in SSA have generalized HIV epidemics with significant numbers of their people living with HIV (PLHIV) on ART. South Africa alone has nearly five million people on ART [1]. As a consequence, HIV clinics across the region tend to be overcrowded and heavily congested. Patients often endure long waiting times and spend a substantial amount of their income on transport to clinics for regular reviews [2].

To ease pressure on over-burdened health systems and to help meet the escalating demand for ART, the World Health Organization (WHO) and major donors such as the President’s Emergency Plan for AIDS Relief (PEPFAR) and the Global Fund to Fight AIDS, Malaria and Tuberculosis (Global Fund) have endorsed ‘differentiated service delivery’ (DSD), which is a novel adaptation to traditional HIV service delivery. DSD contrasts with the traditional HIV care ‘one-size-fits-all’ approach, that is, undifferentiated to the needs of individual patients. Traditional HIV treatment services are clinic-based, physician-centered and entail fixed monthly visits to clinics regardless of clinical need. For instance, Uganda is currently implementing four DSD models which include; i) Fast Track Drug Refill (FTDR) that entails receiving 3–6 monthly medication refills freed from clinical review, ii) Community Client-Led ART Delivery (CCLAD) where voluntary groups of 6–12 patients rotate in picking up medication refills from facilities iii) Community Drug Distribution Points (CDDPs) where outreach sites within communities are designated for medication pick-ups and iv) Facility Based Group (FBG) where patients form adherence support clubs. Select providers in Uganda such as The AIDS Support Organization (TASO) piloted the FTDR model prior to WHO’s formal endorsement in 2016 which enhanced uptake by RoC when these models were formally approved in 2017 in Uganda.

Differentiated HIV treatment services entail a reduction in the intensity of contact by patients with the health system from monthly to quarterly or even six-monthly visits, particularly for those who are clinically stable [3–9]. The WHO, in its landmark 2016 guidance on HIV service delivery under chapter 6, approved DSD based on evidence from clinical trials which demonstrated that viral load suppression under DSD models were as good as under traditional care [2, 3, 10].

DSD holds multiple advantages for the health system such as reducing health worker workloads due to greater appointment spacing afforded to patients stable on ART. From the perspective of patients, DSD entails savings in time and transport through reduced frequency of visits to clinics [3–9]. During Covid-19 lockdowns, community-based HIV care platforms proved a life line for dispensing antiretrovirals such as outreach sites for medication pick up, including home-based delivery in the face of bans on public transport [11–15].

Several countries in SSA have been implementing DSD since the WHO’s landmark 2016 guidelines were published, and previous studies have focused on implementation barriers to DSD roll-out [5, 7] and patient and provider perspectives on the acceptability of these novel HIV service delivery approaches [5–7]. However, there is a paucity of data on the adoption (and non-adoption) by different countries of these novel HIV treatment service delivery approaches.

**Fig. 1:**
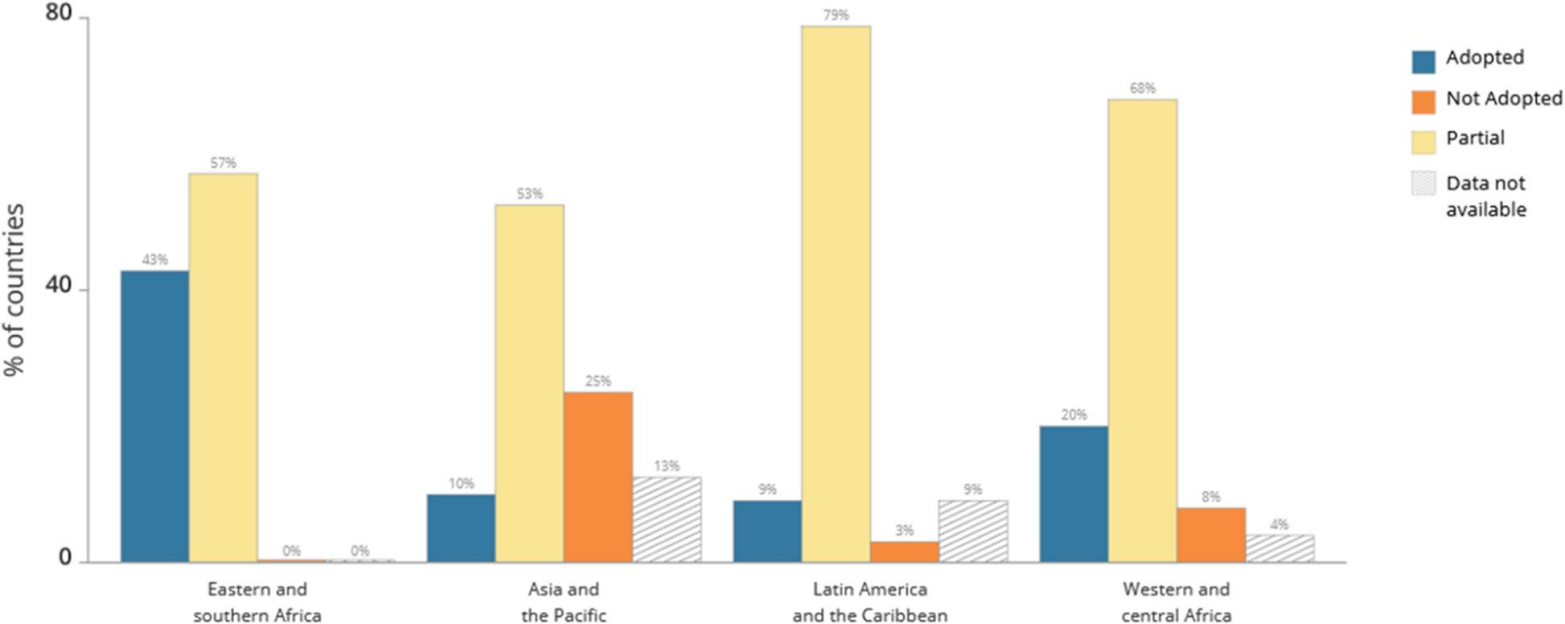
Policy adoption by UNAIDS region

**Fig. 2:**
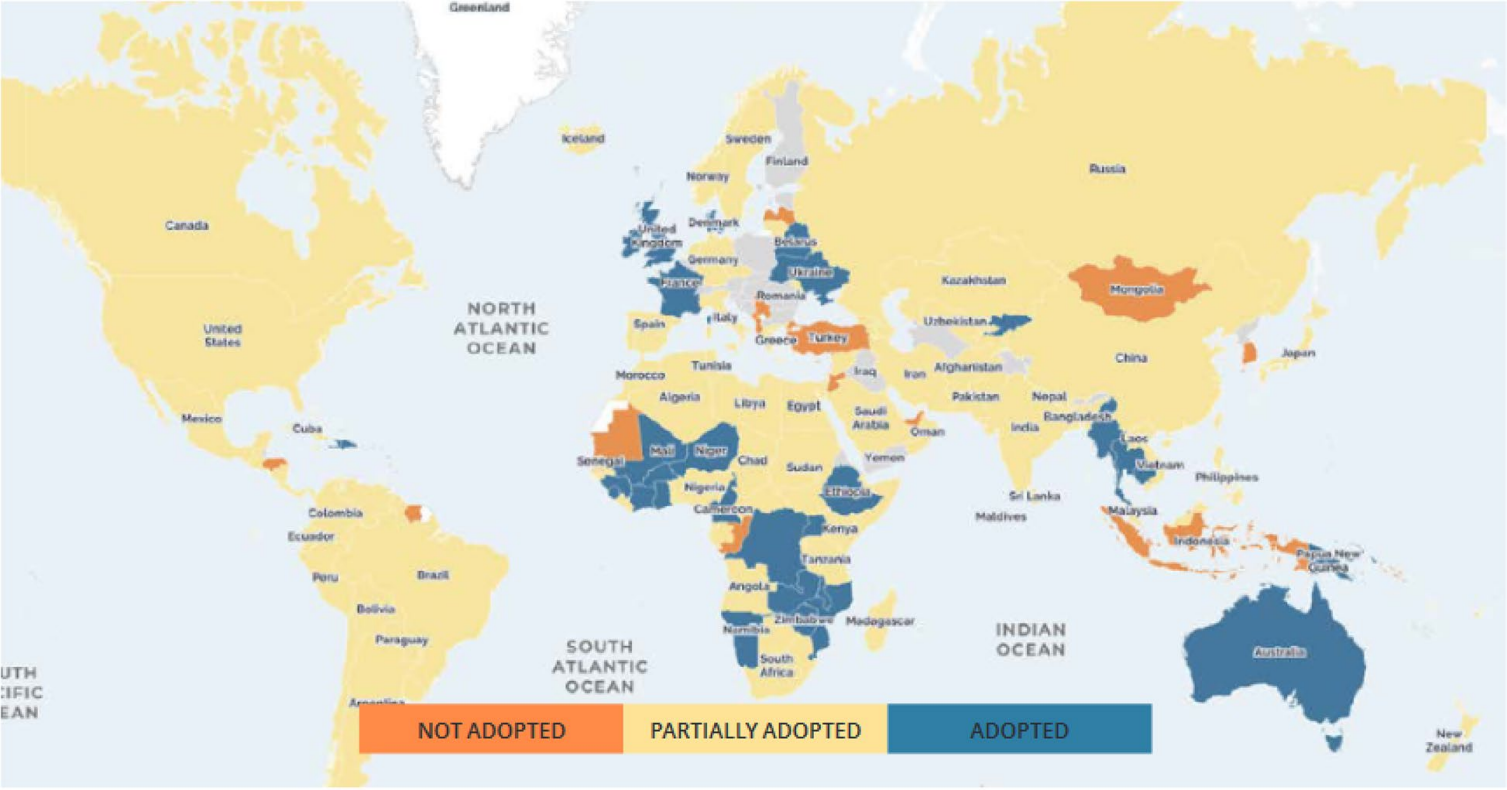
Policy adoption across the globe

This study is partly prompted by findings from the HIV Policy Lab’s global report of March 2022 [16] which reveals substantial variations in the uptake of differentiated HIV treatment services across countries, and regions of the world based on 2021 data (Fig. 1). Although this report shows that policy adoption of differentiated HIV treatment services is uneven across the globe based on 2021 data (Fig. 2), including in countries of Eastern and Southern Africa with high HIV burdens, there is little research seeking to understand the factors underpinning these variations in programmatic uptake across the globe. Expanding the evidence base around facilitators and barriers to programmatic uptake in countries with a high HIV burden, especially those in resource-limited settings. This is valuable to major global HIV funding agencies such as PEPFAR and the Global Fund, who are interested in strategies for the further scale-up of DSD globally. For national-level HIV program managers in high burden countries, evidence is needed on drivers of DSD uptake for policy planning and programmatic interventions as well as in understanding demand-side factors from the perspective of recipients of HIV care (RoC) [1–5, 10]

We utilize an implementation research lens [17] to understand drivers of ‘early adoption’ [18] of differentiated HIV treatment services, with Uganda as a country case study. The implementation science literature distinguishes between ‘early adopters’ and ‘late adopters’ of health care innovations [18]. Rogers (2003) introduced the notion of five categories of adopters (Innovators, Early Adopters, Early Majority, Late Majority, and Laggards) [19]. Early adopters of innovation play a critical role in the successful spread of the innovation by legitimizing the adoption of the innovation and/or providing evidence of its effectiveness’ [20]. In this paper, we utilize Uganda as a case study to understand drivers of ‘early adoption’ of differentiated HIV treatment services with regard to programmatic uptake.

## Methods

### Research design

We adopted a qualitative case study research design [21–23]. As Yin (1994) suggests, ‘Case studies are in-depth investigations of a single instance of a phenomenon in its real-life context’ [24]. We used Uganda as a country case-study to offer explanatory insights into understanding drivers of ‘early’ programmatic uptake of differentiated HIV treatment services. Yin [21], and Stake [25] justify the use of a single case for an in-depth understanding of a unique case thereby providing ‘a nuanced, empirically-rich, holistic account of specific phenomena’. Previous studies have conducted single country case studies [26–29].

### Case selection

Uganda was purposively selected because it adopted WHO’s 2016 landmark international guidelines on differentiated HIV treatment services in its national HIV treatment guidelines the same year they were released [7]. Secondly, Uganda is often considered a model of DSD delivery and uptake and countries such as Ghana have undertaken visits to the country to learn from its DSD implementation experience [30, 31]. The HIV Policy Lab data of 2022 reveals that Uganda is one of the countries which has adopted all three salient features of differentiated HIV treatment services which include community ART distribution, maximum flexibility for clinic visits and multi-month dispensing [18]. Select providers in Uganda piloted select DSD models (such as FTDR) prior to their formal endorsement by the WHO in 2016 [32]. Providers in Uganda such as The AIDS Support Organization (TASO) had already developed guidelines for less-intensive HIV care models which informed Uganda’s 2017 implementation guidelines for DSD.

Uganda’s resource-constrained setting is similar to that in other countries in SSA and the implementation research ‘lessons learned’ approach is likely to have relevance beyond Uganda. Gilson and colleagues [23] have highlighted the utility of case-studies in enabling ‘analytic generalizability’ beyond the individual case(s).

### Theoretical orientation

This study was underpinned by the Consolidated Framework for Implementation Research (CFIR) framework [17]. The CFIR is a ‘meta-theoretical’ analytical framework which is informed by a robust systematic review of the facilitators and barriers to the uptake of healthcare innovations [33]. The framework comprises of 39 constructs that are categorized under five domains: (1) *intervention characteristics* (perceived effectiveness, quality, adaptability, complexity); (2) *outer setting* (external policies and legal frameworks); (3) *inner setting* (organizational priority, implementation climate, leadership engagement); (4*) characteristics of individuals* (patient preferences, patient beliefs, level of income) and; (5) *process* (planning, stakeholder engagement, executing). The five CFIR-derived domains guided this study in multiple ways; it was used to categorize our study participants, it provided a structure for our interview topic guides, it guided our data analysis and in the overall interpretation and synthesis of study findings.

### Study population and sample selection

In line with the adopted analytical framework [17], we purposively selected actors at different levels of the health system in Uganda (macro, meso, micro) [34] (Table 1). At the macro (or national) level, we sought informants with mandates for HIV policy development and programmatic oversight. These included national-level HIV program managers at the Ministry of Health headquarters. Uganda is heavily dependent on international assistance in its national HIV response [35]. External donors, particularly PEPFAR, are influential in setting policy on HIV programming such as setting targets for HIV epidemic control [36]. Hence, we sought out representatives of PEPFAR and its implementing organizations to gain an external donor lens on policy adoption of differentiated HIV treatment services and programmatic uptake. At sub-national level, we sought district health team leaders and district health officers (DHOs) who provide district HIV programming leadership. This was important as Uganda’s decentralized health system means responsibility for service provision has been devolved to sub-national units [37]. At the meso-level, we elicited provider perspectives on the operational context underpinning uptake of differentiated HIV treatment services at the facility and community levels. At the micro-level we included recipients of HIV care (RoCs) among our participants to gain a ‘demand-side’ dimension [38] on the five DSD models on offer in Uganda.

**Table 1:** Category of participants

Respondent type	*N* =
National-level HIV program managers	18
District Health Team leaders	26
HIV clinicians	36
Representatives of regionally-based PEPFAR Implementing Partners (IPs)	11
**Focus Group Discussions**	6
Recipients of HIV care	60

#### In-depth interviews

Between June and September 2020, we conducted sixteen in-depth interviews (IDIs) with national-level HIV program managers at the Ministry of Health headquarters and five district health officers (DHOs) in Eastern Uganda. We held four IDIs with representatives of PEPFAR implementing organizations. We interviewed twelve HIV clinicians to understand provider-level perspectives on facilitators and barriers to uptake of differentiated HIV treatment models. Table 1 shows the category of participants we enrolled in the study. An in-depth interview guide was constructed around the five deductive themes proposed by the CFIR framework (Characteristics of the intervention, Inner setting, outer setting, individuals, process of implementation). The in-depth interview guide was approved by Mildmay Uganda Research Ethics Committee(MUREC). The IDIs were conducted by the first author who has extensive experience in qualitative health services research [39]. The first author was assisted by two research assistants who operated the recorder and took notes during the proceedings. The interviews were conducted in the English language. The face-to-face interviews were conducted on-site at the offices of participants at their workstations.

#### Focus groups

We aimed to understand the perspectives of RoC around trends in the uptake of either community-based or less-intensive facility-based models of care. We sought the collective experiences of RoC as a group rather than as individuals. To this end, we conducted focus group discussions seeking to understand the ‘demand-side’ dimension [38] of the uptake of these novel HIV care models.

We included RoCs who were eighteen years of age or older. We enrolled RoCs who had been in differentiated HIV treatment models for at least twelve months and had substantial experiences in these models. We conducted five FGDs ensuring that we conducted at least one focus group with patients in each of the five differentiated HIV treatment models currently in implementation in Uganda. We had twelve participants in each of our five focus groups (sixty participants). The focus groups were conducted by the first author who was assisted by two research assistants who took notes during the proceedings and operated the recorder. The focus groups were mostly conducted in English but also in Lusoga, the local language spoken at participating facilities in East-central Uganda depending on participants’ fluency in either language. We purposively sampled ROC from East-Central Uganda because it was one of the first regions in Uganda to roll-out DSD models at routine points-of-care. ROC in this part of Uganda were deemed to have substantial experience of HIV care under the varied DSD models models. East-central Uganda is a largely rural and the majority depend on subsistence agriculture for their livelihoods. We selected ROC from Jinja Regional Referral Hospital. The HIV clinic is a stand-alone unit within the larger hospital complex. Jinja is 87 km East of the Ugandan capital of Kampala. The majority of ROC hail from neighboring rural districts and derive a livelihood from subsistence agriculture.

#### Documentary review

An established strength of case studies is the reliance on multiple data collection approaches [21–23]. To this end, we conducted a desk review of documentary evidence relating to the roll-out of differentiated HIV treatment services in Uganda to supplement our core primary data collection. Our desk review was guided by procedures recommended by the WHO [39]. Examples of documents we reviewed include PEPFAR-Uganda country operational plans [40] which outline annual donor HIV programming funding priorities and Uganda’s national implementation guidelines for DSD for HIV treatment of 2017 [41]. In addition, we reviewed relevant websites that helped us construct implementation timelines for the programmatic adoption of DSD and those that highlighted international assistance to Uganda in its policy adoption of differentiated HIV services [30, 31, 42].

### Data analysis

Qualitative data were thematic analyzed [43], using the framework analysis approach [44]. Qualitative data emerging from the in-depth interviews and focus groups were merged during the process of data analysis. Audio recordings from the later were transcribed verbatim into text transcripts (and translated into English where necessary) by two professional transcribers. Based on the framework approach, the data were analyzed in four major steps, although this entailed an iterative process: i) Data familiarization: Interview transcripts were read multiple times by the first author and two co-authors (CK and JD) ii) Development of a coding framework: We used a qualitative data analysis software program during the coding process (ATLAS-ti Center, Berlin). Two authors (HZ, CK) inductively coded the data. The emergent code book was reviewed by two co- authors in protracted peer debriefing sessions. After incorporating feedback from the latter two authors, the emergent code book was applied to all the transcripts generated from the IDIs and FGDs. iii) Abstracting data: The resulting inductively-derived codes were categorized under the five CFIR themes or ‘domains’ (*Process of implementation, characteristics of the intervention, Inner setting, Outer setting, Individuals*). Hence, we used a hybrid model of inductive and deductive theme development [45]. iv) The fourth step in our qualitative data analysis was that of overall interpretation and synthesis involving all the authors. Disagreements in coding/ theme development were resolved through consensus.

## Results

The findings emerging from this study are presented based on the five CFIR-derived domains (*Process of implementation, inner context, outer setting, individuals and characteristics of the intervention*) [17]. The inductively-derived sub-themes are grouped under the five deductive themes above.

### Process of implementation

**Fig. 3:**
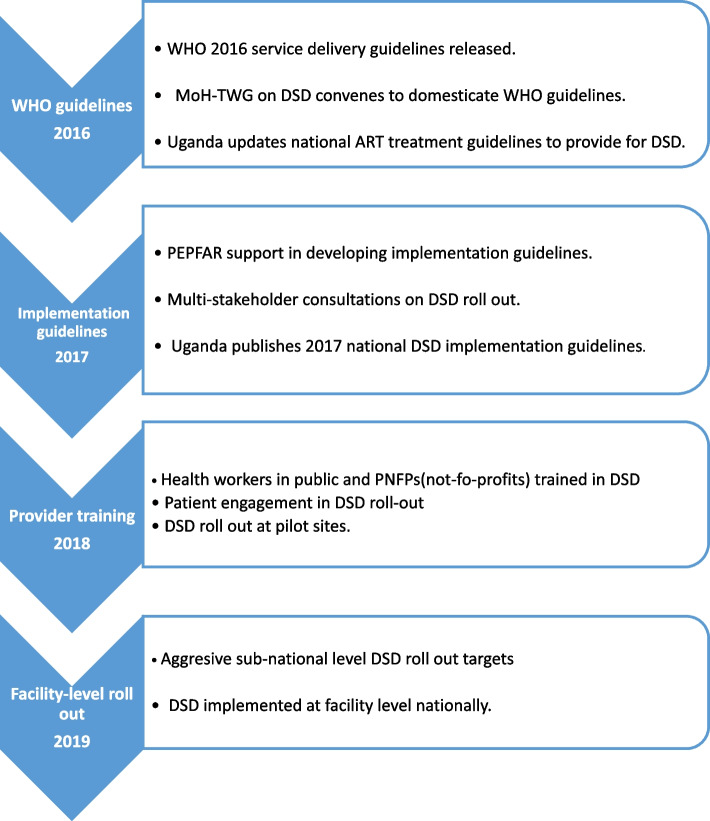
DSD Implementation timelines

Figure 3 shows the timelines involved in the process of programmatic uptake of differentiated HIV treatment services in Uganda which occurred at three levels: (1) national-level (policy adoption) (2), sub-national (programmatic supervision) and (3) facility-level (implementation).

#### National-level policy adoption

National-level informants were emphatic in relaying the notion that Uganda was an ‘early adopter’ of DSD. To demonstrate this point, national-level HIV program managers reported that Uganda adopted WHO’s 2016 global guidelines on DSD in the same year they were released. In 2016, a Technical Working Group (TWG) on DSD was constituted at the Ministry of Health headquarters with a broad membership that co-opted civil society groups representing patient interests. The brief of the TWG was to scrutinize WHO’s global guidelines and develop national DSD guidelines that were context-specific to Uganda and defined which DSD models were feasible to implement given the operational context in the country. Uganda updated its overall national ART treatment guidelines in 2016 to incorporate differentiated HIV services.

‘We began implementation of DSD with the guidelines of 2016 released by World Health Organization When these guidelines were released, Uganda adapted them and like I mentioned, a Technical Working Group sat and decided to develop what is fit for us in our context based on what our situation was like in terms of our HIV Programming. Different countries have categorized their different models, so this is specific to Uganda. You will hear different nomenclatures, as you read a lot but for Uganda this is what we work with’ [National-level HIV program manager]

In 2017, with external support from PEPFAR, national DSD implementation guidelines were unveiled to guide roll out at 474 pilot high-volume HIV clinics in 2018, with countrywide roll-out through 2019. These implementation guidelines provided for two community-based models and three facility-based models. The implementation guidelines also provided a detailed ‘roadmap’ for adopting differentiated HIV treatment services and delivery mechanisms, including patient ‘differentiation’ clarifying which patients were eligible for which models and the formation of facility-level DSD committees.

#### Community engagement

A salient feature of DSD adoption and implementation in Uganda was the protracted involvement of recipients of HIV care in the design and roll-out of Uganda’s five DSD models which enhanced client uptake of these innovations in HIV treatment delivery. Patients were involved right from the inception with their representation on the national DSD Technical Working Group at the Ministry of Health in 2016, in the development of national implementation guidelines in June 2017, and their involvement in on-site evaluations of the quality of DSD services offered at pilot sites in Uganda.

‘One of the facilitators of adoption of evidence on DSD was about engaging with recipients of HIV care and their leaders. We have tried to engage patients all the way from policy to program implementation. Patients were represented on the Technical Working Group which domesticated WHO’s 2016 guidelines to our context. Stakeholder engagement has been key. Patients through their leaders give us feedback as the Ministry of Health. How can we do this better? What are the voices of the people (patients)? They actually tell us’. [National-level HIV program manager]

Beyond development of national DSD implementation guidelines, national-level HIV program managers indicated that dissemination of the approved guidelines was fundamental in moving from policy to routine practice. With funding from PEPFAR they were able to conduct multiple planning meetings for national roll-out of DSD between 2017 and 2018 at the national-level with HIV services managers of public and private providers. At the sub-national level, regionally-based PEPFAR implementing organizations were mandated to spearhead roll out at the level of districts given Uganda’s decentralized health system set up whereby overall responsibility for social services provision has been devolved to sub-national units.

‘We have disseminated these (DSD) guidelines at different levels from the national, regional, up to the facility levels and I know that one or two people received those guidelines at each of the levels’ [National-level HIV program manager]

Between 2017 and 2019, Uganda embarked on an ambitious programme of health worker (HW) training in DSD delivery. These trainings were mainly conducted at sub-national level where regional trainings were convened, and facility-level HIV clinicians were invited. At these meetings clinicians were sensitized on national DSD implementation guidelines of 2017 such as selecting a DSD focal person at each facility and offered practical trainings in aspects such as patient differentiation selecting patients who were stable on treatment and assigning them to less-intensive models such those who could access their refills in the community. In addition, on-site supervisions were conducted as part of the evaluations of differentiated HIV treatment services.

Recipients of HIV care were involved in providing feedback on pilot DSD services. Patient leaders have been engaged in support supervision of DSD at the facility-level to ensure that quality is offered and implementation is aligned with policy and intervention design [National-level HIV program manager]

### Inner context

#### Institutional history

Our analysis reveals that country context is relevant in understanding early policy adoption of evidence on DSD. Uganda has a generalized HIV epidemic and has one of the greatest HIV burdens in Eastern and Southern Africa [46]. Uganda’s institutional history of high-level support for a national HIV response dating back to the early 1990s served as a conducive backdrop for the expedited policy uptake and implementation of WHO’s global guidelines on DSD for HIV treatment [47].

‘We have one of the oldest HIV treatment programmes in Africa. We have been doing HIV programming since 2004. Uganda is one of the countries I think that is at the forefront of implementing DSD and I would like to say that several countries are flying into Uganda to see how Uganda is doing DSD. We were one of the first countries to implement this and we are learning by doing.’ [National-level HIV program manager]

#### ‘DSD is not new’

Our interviews with national-level HIV program managers revealed that an important enabler of policy transfer between international and national levels of DSD for HIV treatment in Uganda was the country’s long-standing experience of implementing innovations in HIV treatment services due to its high HIV burden, the associated challenges of overcrowding at routine points of care within an overburdened health system. Participants frequently mentioned that Uganda had a head start in implementing adaptations to traditional HIV service delivery models given its decades-old experience of attempts at mitigating health system constraints in the face of escalating demand for HIV treatment.

‘We were already doing differentiated HIV care in Uganda even before the term was coined as DSD but as a country, we did not have a systematic way of implementing these innovations in HIV care. What made policy adoption of DSD easier in Uganda is that we were already implementing these innovations such as appointment spacing long before they were baptized as such. The fact is that DSD is not new, and what is new is the nomenclature. Uganda is one of the countries that is a leader in implementing DSD and I would like to say that several countries are flying into Uganda to see how Uganda is doing DSD. We have tried to engage all the way from policy development to implementation’ [National-level HIV program manager].

#### Provider implementation experience

It emerged that some of the evidence underpinning WHO’s global guidelines on differentiated HIV treatment services derived from Uganda’s experience as a facilitator of programmatic uptake. Some private providers in Uganda such as The AIDS Support Organization (TASO) were already implementing innovations such as community drug distribution points (CDDP) and Fast Track Drug Refills (FTDR) before the models were formally endorsed by the WHO in 2016. Participants revealed that uptake of DSD was enhanced by Uganda’s participation in pilot studies assessing patient outcomes on less-intensive HIV care models for the clinically stable.

‘In Uganda we were already doing longer appointment spacing for stable patients many years ago. In fact, some of the evidence underpinning DSD came from Uganda. Some providers like TASO piloted these models for many years before WHO endorsed them in 2016. For us as TASO, it has been a walk over because before the DSD models started we had already started implementing them. So it has just been a revision. Already our clients are well versed and they know what to do and also our health workers are fully trained’ [HIV clinic manager, Not for profit provider, Eastern Uganda]

#### Covid-19 and acceleration of DSD for HIV treatment

In March 2020, Uganda announced Covid-19 ‘lockdown’ measures that included a ban on public and private transport. This effectively impeded facility-based access to drug refills for the 1.4 million Ugandans enrolled on HIV treatment across the country. In response, the Ministry of Health swiftly announced emergency policy guidelines for enabling access to HIV medication refills during lockdown restrictions. The policy measures entailed an aggressive expansion of decentralized medication refill deliveries at outreach sites within the community [48]. The guidelines which were unveiled, extended the interval of antiretroviral therapy refills from three to six months for clinically stable patients and expanded the eligibility for multi-month dispensing to unstable patients who were permitted an unprecedented three-months medication refill.

On their part, a range of providers such as TASO leveraged existing community-based models of service delivery such as the CDDP model, which was scaled-up to maintain continuity in access to refills.

‘CDDP was a model that was especially helpful during Covid-19 lockdown. What made life simple for us in TASO is that we had started DSD models much earlier and it made life easy for clients because the health workers meet clients at the community distribution points (CDDP) venue; where they were picking their drugs. At TASO, we have been doing multi month dispensing for stable clients. We have now drugs to give them for six months for stable clients and three months for unstable clients

There was accelerated implementation of home-based HIV medication refills delivery. Not-for-profit providers such as TASO mobilized vehicle fleets for distribution of refills on patients’ homes deep within quite remote rural communities.

‘Covid-19 came in as an advantage. We would do home-to-home drug delivery so we took the drugs to their homes. For those we were unable access in their homes we could make an arrangement for them to be in a nearby place in their community. [HIV programme manager, Eastern Uganda].

At sub-national level, district health teams sourced funding from regionally-based PEPFAR implementing organizations in Eastern Uganda for mobilizing vehicle fleets and fuel for distributing medication refills at designated points in the community.

‘We engaged our regional implementing partner (RHITES- E) and they were kind enough to offer some vehicles and we used them to reach people in their communities where they were stuck due to bans on public and private transport. We took medicine to about 2,000 people to some designated outreach points in the community by bolstering and strengthening our CDDP (community drug distribution points) model. We did this with the aid of a mobile brigade of health workers’ [District Health Officer, Eastern Uganda].

### Outer context

#### International assistance in DSD uptake

In several PEPFAR ‘focus countries’, including Uganda, PEPFAR seconded Technical Advisors to Ministry of Health headquarters to spearhead country wide roll-out of DSD. PEPFAR typically provides salary support to key officials at the Ministry of Health headquarters with governance mandates for HIV epidemic control and for whom PEPFAR targets such as domesticating DSD evidence in national policies is key programmatic target [30]. Hence, the presence of a considerable number of DSD ‘program champions’ at the highest level in the Ugandan Ministry of Health was a contributory factor in early policy adoption and subsequent implementation [40].

In-depth interviews with national-level HIV program managers in Uganda revealed the nature of donor support they received from PEPFAR for policy adoption of differentiated HIV care besides annual program budgets and targets described in the PEPFAR Country Operational Plans (COP) from 2018–2021.

‘PEPFAR has been consistent in prioritizing the uptake of differentiated HIV services in Uganda. PEPFAR has provided millions of dollars in annual HIV programming budgets for Uganda for more than five years. PEPFAR strongly believes in the potential of DSD in reducing burdens on overwhelmed health systems and in improving the quality of HIV care’ [National-level, HIV program manager].

Participants reported that PEPFAR provided program funding for convening Technical Working Groups at the Uganda Ministry of Health headquarters with the aim of scrutinizing WHO’s 2016 landmark global HIV treatment guidelines with a view to domesticating them in Uganda’s national ART treatment guidelines. National-level HIV program managers reported that they assessed the WHO’S 2016 guidelines on DSD roll-out and teased out components that were contextually relevant and feasible to implement in Uganda. At sub-national level, PEPFAR provided multi-year funding and programmatic targets to its regionally-based implementing organizations to spear head DSD roll out at district level [49–51].

Documentary evidence further revealed that besides PEPFAR support, Uganda benefited from substantial philanthropic aid from the Bill & Melinda Gates Foundation for policy adoption of DSD through Columbia University’ CQUIN (the HIV Coverage, Quality Improvement Network) entailing a multi-country ‘community of practice’ of more than eleven countries in Eastern and Southern Africa [34]. Through CQUIN, Uganda received technical support to ‘adopt, implement, and expand effective DSD models by enabling experience sharing, south-to-south exchange, and collaborative problem-solving, and by providing targeted, demand-driven technical assistance and support’ [52]. Gates Foundation multi-million dollar grants enabled Uganda and other high burden countries to receive technical assistance and support from global experts in the national scale-up of DSD right at inception or Uganda’s adoption of implementation guidelines in June 2017 to date [53].

Gates Foundation support also included tracking progress on programmatic adoption of DSD through specified metrics for assessing progress on programmatic uptake of DSD evidence via a ‘dashboard’ for measuring progress on DSD implementation. The dashboard assesses the maturity of national DSD programs and overall uptake based on thirteen domains that included presence of a scale-up plan, policies in place, community engagement and training of health workers [54].

### Characteristics of individuals

#### Patient preferences driving model uptake

Overall, patients indicated a preference for facility-based DSD models due to a desire for privacy and avoidance of inadvertent HIV sero-status disclosure. In the focus groups, patients appreciated the convenience that accrues from some models such as Fast Track Drug Refill (FTDR) which entails visits to facilities that are freed from clinical reviews for stable patients. In addition, patients extoled the benefits that accrue from regular visits to facilities such as comprehensive medical care in the event of opportunistic infections which they perceived as difficult to track when one is enrolled in less-intensive HIV care models. Patients indicated that HIV-related stigma was a fundamental barrier to enrollment in community-based models.

‘First of all, I fear rumor-mongering amongst members in these patient groups. I imagine going back in my neighborhood and finding my group member gossiping about me in the village and telling everyone about how I am HIV positive. It was not easy for me to join a (CCLAD) group because I don’t want people to gossip about me in the village’ [Recipient of HIV care, Eastern Uganda].

At the provider-level, HIV clinicians offered explanatory insights into understanding the variable uptake of DSD models in Uganda, particularly the lag in adoption of community-based models despite a heightened interest during Covid-19 lockdown restrictions. It emerged that select community-based ART delivery models such CDDPs were perceived as relatively expensive to operate in terms of the required operational expenses such as with regard to the needed vehicle fleets and fuel for transporting medication refills to outreach sites deep inside rural communities [26, 28]. This may partly explain the lag in adoption of these models in some countries.

‘We have some community-based models that are expensive such as community drug distribution points (CDDPs) because we have transport costs borne by the facility. I think that the most practical DSD models remain the facility –based ones. It is not sustainable going to these communities. You are able to deliver these medicines in communities now just because there is external donor funding but time is going to come when there is no funding’ [HIV clinician, Northern Uganda].

National-level HIV program managers in Uganda also revealed that HIV-related stigma is a fundamental impediment in the roll-out of community-based HIV treatment models which could provide further contextual insight into understanding the relatively low uptake of community models.

‘I wish to highlight the issue of HIV-related stigma, I think that as we plan on implementing community -based models of care, it is incumbent upon us to regard stigma as a key challenge which still exists in our communities. The few on-site support supervisions that I have conducted around the uptake of DSD is we found that clients actually prefer facility-based HIV care. The do not like community models because of stigma’ [Representative of PEPFAR implementing organization, Western Uganda].

### Intervention characteristics

#### Perceived effectiveness

There was broad consensus across participants that differentiated HIV treatment models were evidence-based which they regarded as a facilitator of acceptance among providers. Uganda’s participation in the clinical trials and the broader evidence that underpinned WHO’s 2016 global treatment guidelines was frequently cited as an enabler of programmatic uptake. WHO’s formal endorsement of DSD was perceived by national-level HIV program managers as affirmation that the endorsement was informed by rigorous processes.

‘WHO goes through a series of processes and one of them is looking at evidence. Evidence was collected from different countries such as South Africa, Malawi and Zambia. By the time it is put into a global treatment guideline it implied that WHO was confident that DSD was evidence-based. You know it is backed by science. So already there has been demonstration that these models actually work and which is why we selected certain models that work well in our setting’ [ National-level HIV program manager].

Representatives of providers, such as from TASO, indicated that they had piloted some community-based models including the community drug distribution model prior its policy adoption in global and national treatment guidelines. Other not-for-profit providers indicated that they had experimented with models, particularly that of appointment spacing even before it was formally adapted by the WHO in 2016 and that from their operational experience, patient outcomes were not inferior to standard care.

#### Patient demand for less-intensive HIV care models

Right from the outset, the design of DSD models was meant to be tailored to the preferences of individual patients and this was identified as an enabler of model uptake in Uganda. Recipients of HIV care in rural settings expressed satisfaction and preference for less-intensive HIV treatment models that resulted in savings in transport and time off work. The demand for community-based models was particularly strong for PLHIV who are based in rural settings who would otherwise have to make monthly trips to urban centres to collect medication refills.

‘Basically, I was overburdened by the heavy expenditure on transport to come to the(HIV) clinic every month and the long distance from my home. I often came late to the clinic and it was always overcrowded. When they said we could form patient groups (CCLAD), I got six of my colleagues and we formed a group. We now share transport costs and alternate in picking our medication refills so that we don’t get tired of coming to clinic’ [Recipient of HIV care, Eastern Uganda].

On the other hand, patients in urban settings revealed preference for facility-based models particularly the Fast Track Drug Refill (FTDR) model which offered them better privacy and convenience.

‘Patients are aware of community models but the majority have internalized stigma. They fear they think their colleagues will gossip about them in their neighborhoods so some people decide not to join the groups. Personally I prefer to come to the facility and pick my drugs. I spend less than ten minutes yet I used to spend the whole day. Now I have ample time to go and man my business’ [Recipient of HIV care, Eastern Uganda].

## Discussion

Utilizing the CFIR framework’s five domains, we explore factors contributing to early adoption of evidence on differentiated HIV treatment services in Uganda. These include a decades-old HIV intervention implementation experience, having a high HIV burden which prompted innovations in HIV treatment delivery, receiving substantial external aid and technical support for policy uptake, protracted engagement of patients in policy planning and program implementation, accelerated uptake of community-based models owing to Covid-19 ‘lockdown’ restrictions and the perceived effectiveness of DSD models due to participation in the clinical trials that underpin WHO guidance on DSD.

Although a few of the emergent factors are context-specific to Uganda, such as its long HIV programming experience, our findings provide valuable implementation research lessons on the processes involved in policy adoption of DSD especially in countries with a high HIV burden. These lessons include the key role of Technical Working Groups in domesticating global guidelines and fleshing out national DSD implementation guidelines, and implementation strategies including high-level health ministry buy-in, protracted patient engagement to enhance model uptake, and devising metrics for measuring DSD uptake progress in programmatic adoption. Our findings provide empirical insight into understanding variations in programmatic uptake of WHO guidelines on differentiated HIV treatment services which have been identified by multiple studies [4, 6, 9] and more recently revealed in the HIV Policy Lab’s global report of 2022 [16].

Previous studies have identified implementation barriers in the national scale-up of differentiated HIV services in countries that include South Africa [55], Uganda [4], Malawi [56] and Zimbabwe [5] while others have focused on reporting patient preferences of DSD models [57–59]. The unique contribution of this study is in providing an in-depth country case study of programmatic uptake of these novel HIV delivery approaches thereby contributing to an understanding of the drivers of ‘early adoption’ and the implementation strategies needed for promoting programmatic uptake which is a global health priority recently recognized by Nathana Ford and colleagues [60]. Utilizing Uganda as a case study, we contribute to answering the call for contributing to a better understanding of *how* and *why* substantial variations in DSD uptake across the globe persist despite the wide dissemination of WHO’s landmark 2016 guidelines on how to domesticate this evidence in the national policies of member countries [16].

Our case study sheds light on the impact of the Covid-19 pandemic on the accelerated uptake of differentiated HIV care models, and their adaptation in light of ‘lockdown’ measures. Grimsrud and colleagues have highlighted the imperative of the Covid-19 in the accelerated adoption of especially community-based ART delivery models [12, 14]. Studies report that ‘lockdown’ measures accelerated the uptake of community-based ART distribution and extended multi-month dispensing in particular [61, 62]. It is important to point out that the policy gains registered during Covid-19 ‘lockdown’, especially around extending refill periods and appointment spacing should be institutionalized and sustained beyond the pandemic phase [11–14]. Furthermore, the Covid-19 pandemic demonstrated the utility of differentiated HIV care during ‘lockdown’, particularly community-based models, including in low-burden countries [61, 62]. Further research is warranted to understand the potential for scaling-up these innovations in ART delivery post-Covid-19 such as understanding the cost-effectiveness of home-based ART delivery which is not adequately understood [63].

In this case study we highlight the role of international assistance in the early adoption of DSD in Uganda. Although a previous analysis by Carbaugh and colleagues [64] found that PEPFAR ‘focus countries’ have closer policy alignment to differentiated HIV services in their national policies when compared to non-PEPFAR countries, in this study we shade a light on the specific nature and type of donor support received such as salary support to DSD Technical advisors at Ministry of Health headquarters in Uganda and funding the development of national implementation guidelines for differentiated HIV services of June 2017.

Several studies have already reported patient preferences for facility-based HIV care over community based platforms [57–59]. A range of explanatory insights have been unearthed in our study that include HIV-related stigma and patient preference of facility-based care due to opportunities for the management of opportunistic infections during facility visits, as well as the need for regular ‘personal touch’ from health workers [59]. The delay in embracing broader DSD models such as multi-month dispensing and community-based ART distribution could be partly due to the implementation barriers experienced in early-adopter countries such as South Africa and Uganda in terms of supply chain capacity constraints in implementing multi-month dispensing [55, 59].

The CFIR framework was helpful in unraveling, especially, the outer setting and inner context drivers of Uganda’s early adoption of differentiated HIV treatment services which afforded us a well-rounded perspective on programmatic uptake of DSD from a multi-level analysis lens; at the different levels of the Ugandan health system such as at the meso or national level, at the sub-national level, at the meso or provider level and at the individual level of patients. In our assessment, some identified drivers, such as Covid-19 lockdown imperatives for accelerated uptake of community models in Uganda, was an attribute that cut across two CFIR ‘domains’ of both inner and outer contexts and that there appeared to be some dynamic interactions in the five CFIR domains such as in the latter case [65]. Relatedly, our analysis suggests that although Rogers’ [19] framework posits that the speed of diffusion stems from individual adopters and arises as a result of their own innate ‘agency’, in the Ugandan case study, diffusion appeared to be driven by both internal and external factors and often there were dynamic interactions in the two.

### Study limitations and strengths

This study had limitations which we wish to acknowledge. We conducted a cross-sectional study where we collected data at one-time point. Perhaps, a longitudinal case study could have been more insightful. Additionally, we did not interview PEPFAR officials at the global level who set donor funding policy for PEPFAR-supported countries across the world which would have yielded a global lens on PEPFAR’s push for policy adoption of DSD in high burden countries. This study has some strengths. These include multi-stakeholder perspectives at different levels of the Ugandan health system such as national-level HIV program managers, sub-national level health system actors, representatives of external donors and community engagement component entailing patient voices. We contribute a country case study that helps in understanding the uneven uptake of these novel HIV service delivery approaches across the globe by identifying the drivers of programmatic uptake from the CFIR lens of ‘inner context’ and ‘outer context’.

## Conclusion

Using the CFIR, we have identified facilitators of early adoption of DSD arising from inner context and outer setting factors in Uganda. Our case study of Uganda contributes implementation research lessons on pragmatic strategies for promoting programmatic uptake of differentiated treatment HIV services in other countries with a high HIV burden particularly those in resource-limited settings.

## Data Availability

The datasets generated during and/or analyzed during the current study are not publicly available due to ethical reasons but are available from the corresponding author on reasonable request.

## Abbreviations

AIDS: Acquired Immune Deficiency Syndrome
ADRs: Adverse Drug Reactions
ART: Anti-retroviral therapy
ARVs: Anti-retrovirals
CFIR: Consolidated Framework for Implementation Research
DSD: Differentiated Service Delivery
MOH: Ministry of Health
PEPFAR: The Presidents' Emergency Plan for AIDS Relief
PLHIV: People Living with HIV
RoC: Recipient of HIV care
SSA: Sub-Saharan Africa
WHO: World Health Organization
